# Supplementary material for: Highly accurate carbohydrate-binding site prediction with DeepGlycanSite
Source: Nat Commun. 2024 Jun 17;15:5163. doi: 10.1038/s41467-024-49516-2 (PMC11183243; doi:10.1038/s41467-024-49516-2)
Supplement: Supplementary file 1 — Supplementary Information [file 41467_2024_49516_MOESM1_ESM.pdf]

Supplementary Information

Highly accurate carbohydrate-binding site prediction with DeepGlycanSite

Xinheng He[1,2,†], Lifen Zhao[1,†], Yinping Tian[1,†], Rui Li[1,3], Qinyu Chu[4], Zhiyong Gu[4], Mingyue Zheng[1,2,4], Yusong Wang[5], Shaoning Li[6], Hualiang Jiang[1,2,4,7], Yi Jiang[7], Liuqing Wen[1, 2,], Dingyan Wang[7,*] & Xi Cheng[1,2,4,*]*

[1]State Key Laboratory of Drug Research and State Key Laboratory of Chemical Biology, Carbohydrate-Based Drug Research Center, Shanghai Institute of Materia Medica, Chinese Academy of Sciences, Shanghai, China

[2]University of Chinese Academy of Sciences, Beijing, China

[3]School of Pharmacy, China Pharmaceutical University, Nanjing, China

[4]School of Pharmaceutical Science and Technology, Hangzhou Institute of Advanced Study, Hangzhou, China

[5]National Key Laboratory of Human-Machine Hybrid Augmented Intelligence, National Engineering Research Center for Visual Information and Applications, and Institute of Artificial Intelligence and Robotics, Xi'an Jiaotong University, Xi'an, China

[6]Department of Computer Science and Engineering, The Chinese University of Hong Kong, Hong Kong, China

[7]Lingang Laboratory, Shanghai, China

Table of Contents

Supplementary Figures 3

Supplementary Tables.. 13

Supplementary Methods 36

Supplementary References.. 43

Supplementary Figures

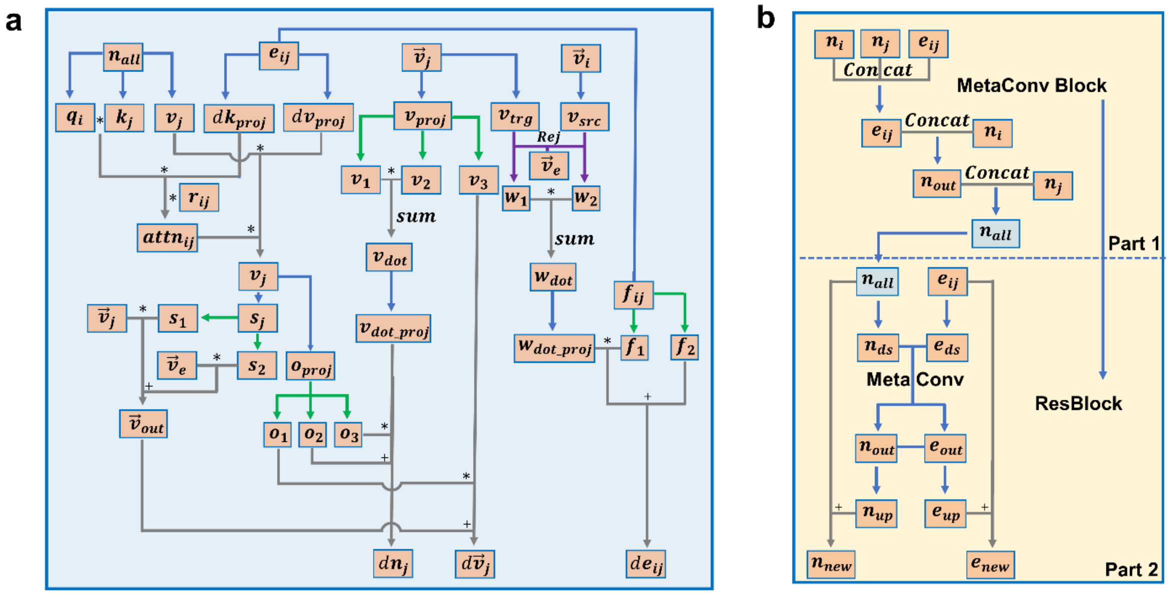

Supplementary Fig. 1 | Information flow within the layers. a The attention layer of ReceptorNet. n is the node feature, e is the edge feature, $\vec{v}_i$ (or $\vec{v}_j$) and $\vec{v}_e$ are node and edge vectors, respectively. Different operations are represented by different arrows: blue arrows are projection, gray arrows are arithmetic computation, green arrows are splitting, and purple arrows are rejection. **b** The information flow in LigandNet with updates of node and edge features.

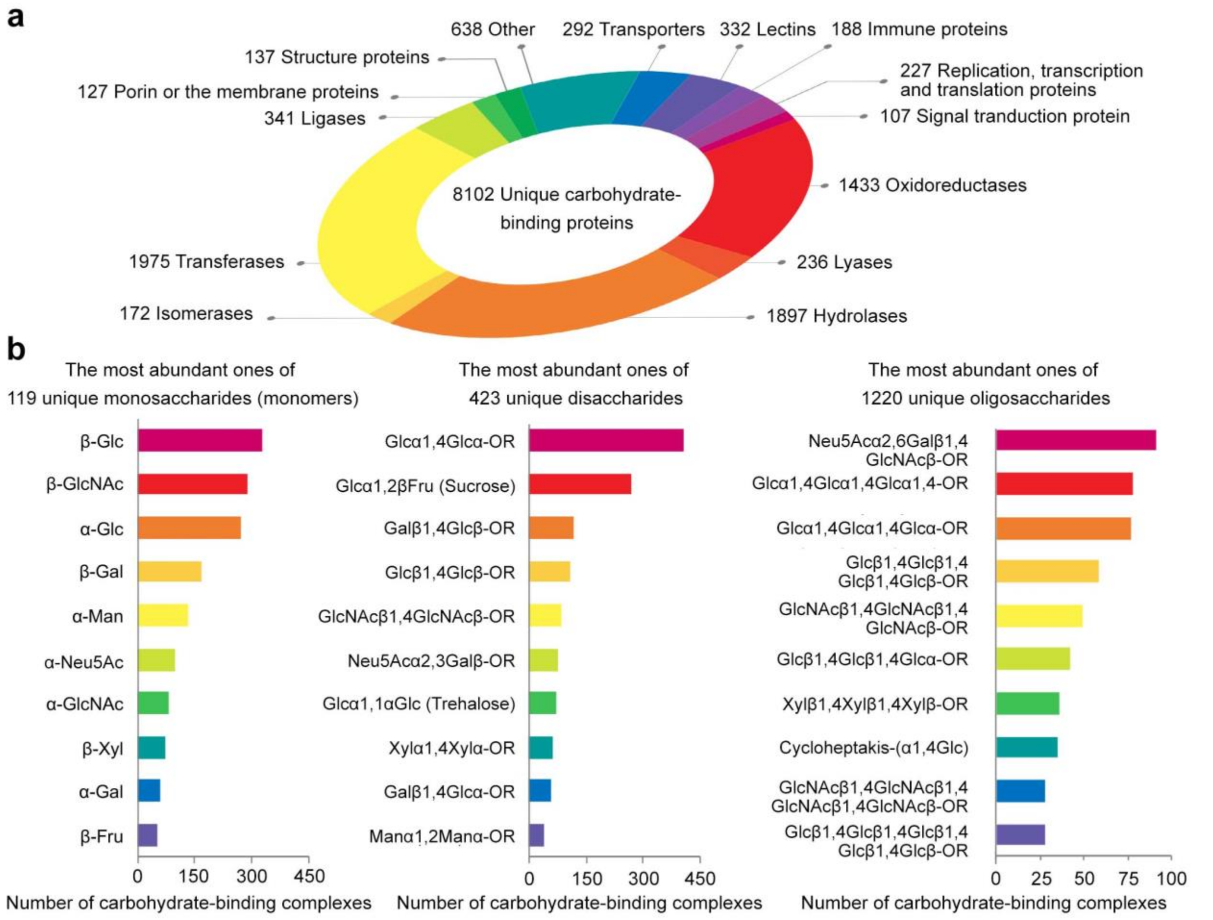

Supplementary Fig. 2 | Diversity of carbohydrate-protein complex dataset. a Families of carbohydrate-binding proteins in our dataset. **b** The most abundant monosaccharides, disaccharides and oligosaccharides in our dataset. Source data are provided as a Source Data file.

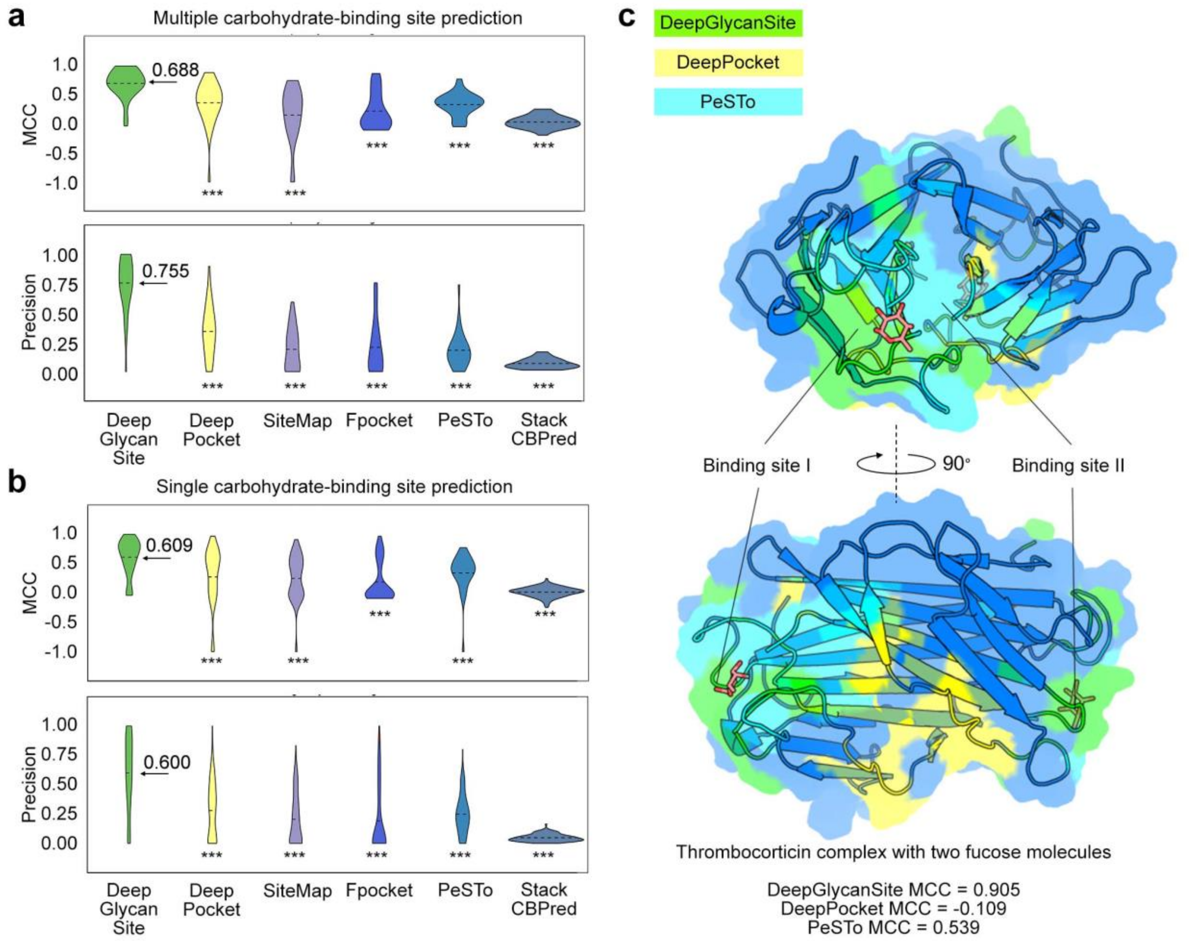

Supplementary Fig. 3 | Comparison of model performance in predicting multiple or single carbohydrate-binding sites. a Average MCC (top) and precision (bottom) of all methods in predicting multiple carbohydrate-binding sites (n = 29). **b** Average MCC (top) and precision (bottom) of all methods in predicting single carbohydrate-binding sites (n = 116). The average values are indicated as solid lines. Two-tailed Mann-Whitney U test is used to determine statistical difference. *** indicates P is less than 0.001. From left to right and top to bottom, the exact P values are 1.5E-5, 2.0E-7, 2.1E-6, 2.7E-7, 8.1E-10, 1.3E-7, 3.0E-9, 2.0E-8, 4.5E-9, 1.2E-9, 6.1E-10, 9.3E-15, 5.7E-17, 1.7E-12, 1.4E-27, 5.5E-14, 1.7E-18, 2.5E-17, 1.0E-16 and 3.4E-24. **c** Predicted carbohydrate-binding sites by DeepGlycanSite, DeepPocket and PeSTo on a protein with multiple carbohydrate-binding sites (PDB code: 7F9G). Predicted

binding sites were mapped on the given protein structures. The carbohydrates are displayed as sticks to indicate the true binding sites. Source data are provided as a Source Data file.

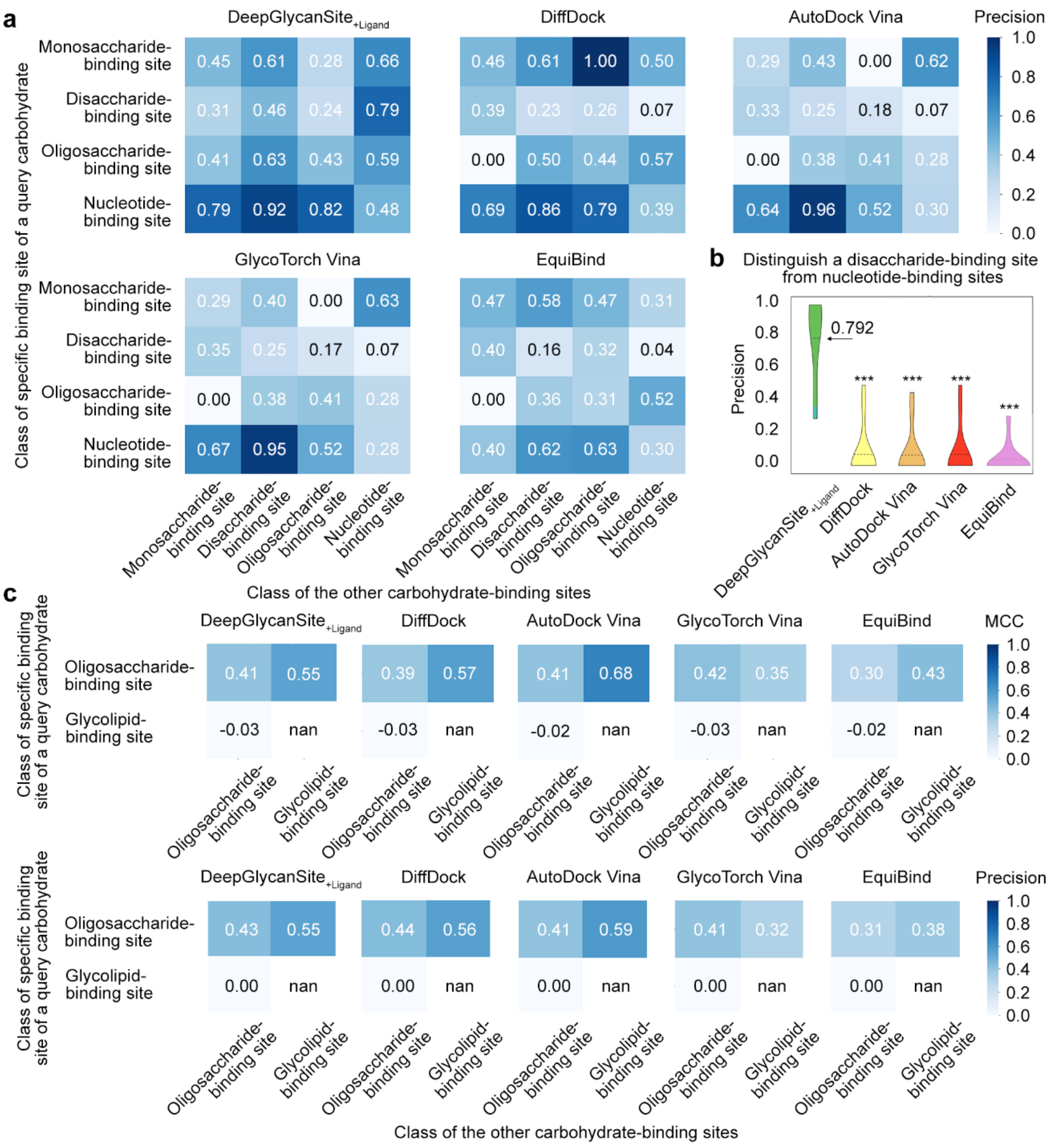

Supplementary Fig. 4 | Specific binding site prediction for a query carbohydrate. a Heatmaps showing the average precision in predicting a specific binding site of a query carbohydrate when the other binding site exists on the same protein. The binding sites are categorized into four classes. **b** Average precision in predicting the specific binding site of a query disaccharide when at least one nucleotide-binding site exists on the same protein. The average values are indicated as solid lines (n = 12). Two-tailed Mann-Whitney U test is used

to determine statistical difference. *** indicates P is less than 0.001. From left to right, the P values are 5.0E-5, 5.0E-5, 5.0E-5 and 2.6E-5. **c** Heatmaps showing the average MCC and precision in predicting a specific binding site of a query carbohydrate when the other binding site exists on the same protein. The binding sites are categorized into two classes. Source data are provided as a Source Data file.

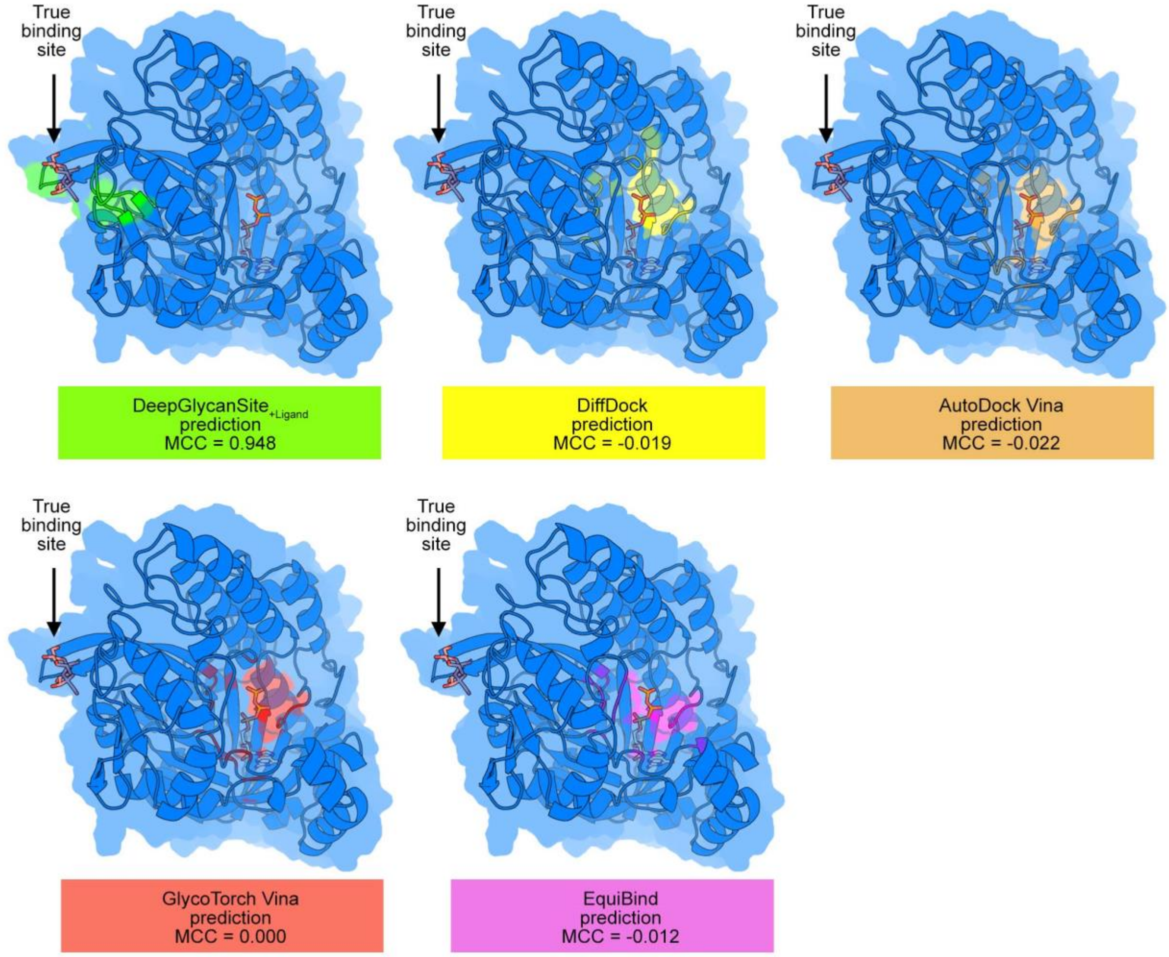

Supplementary Fig. 5 | Predicted specific binding sites of a query disaccharide by all methods on a representative protein binding to two different glycans (PDB code: 8DCD). Protein is shown in cartoon and surface depict. To show the location of the predicted sites, the DeepGlycanSite$_{+Ligand}$ (green), DiffDock (yellow), AutoDock Vina (orange), GlycoTorch Vina (red) and EquiBind (purple) predicted sites were mapped on the given protein structures. The binding disaccharides (salmon) and nucleotides (gray) are displayed as sticks to indicate the true binding sites and the other sites, respectively.

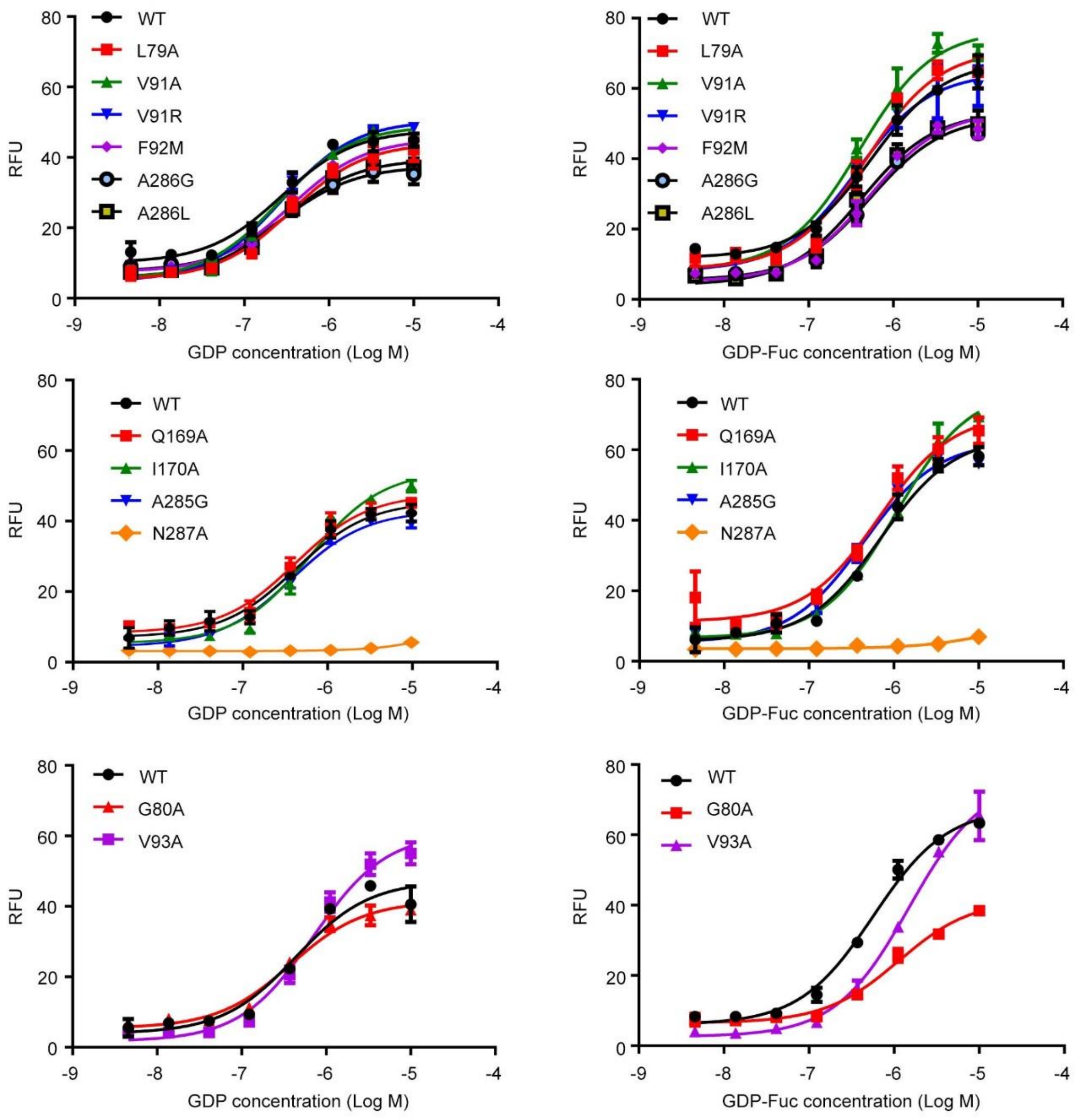

Supplementary Fig. 6 | Calcium mobilization concentration-response curves for GDP or GDP-Fuc in HEK293 expressing P2Y14 wild-type (WT) and mutants. Data are presented with a minimum of three independent biological replicates. The error bar indicates standard error. Source data are provided as a Source Data file.

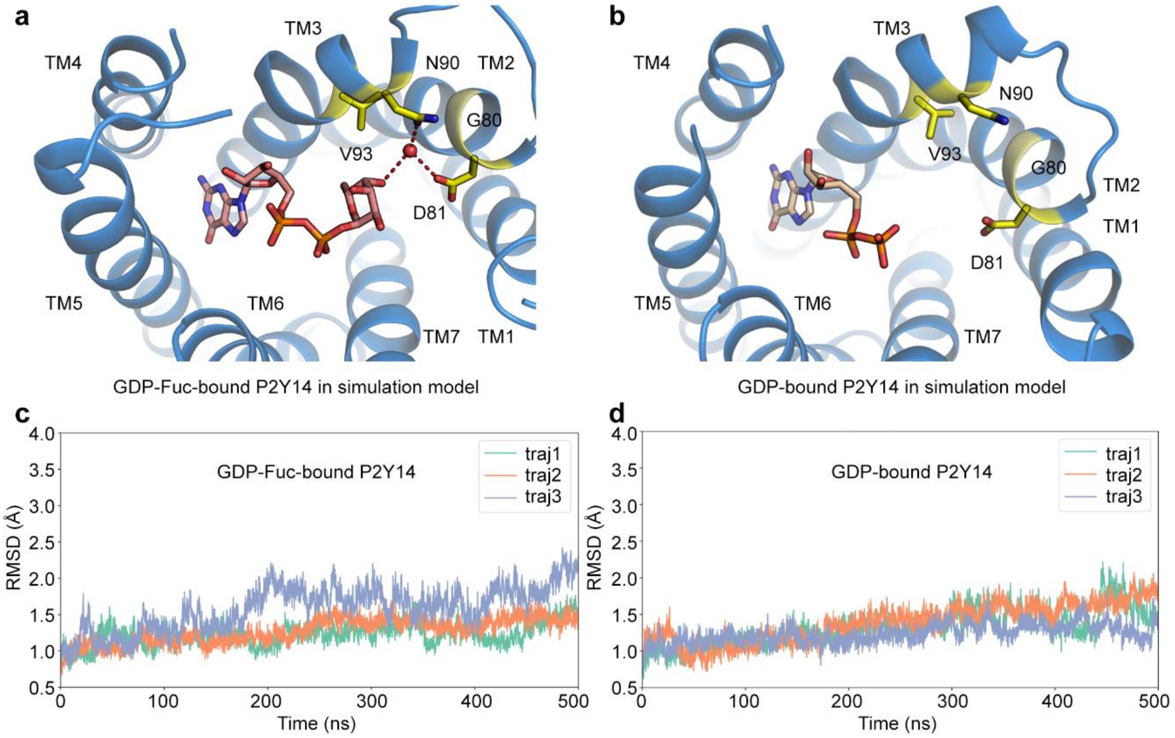

Supplementary Fig. 7 | The ligand binding mode shown by MD models. a, b Representative MD simulation models of the GDP-Fuc-bound (**a**) and GDP-bound receptors (**b**). Ligands and key residues are as shown as sticks. A key water molecule is shown as a sphere. Putative hydrogen bonds are shown as dash lines. Source data are provided in the simulation files. **c, d** The Cα atom root mean squared deviation (RMSD) of transmembrane domains in GDP-Fuc-bound (**c**) and GDP-bound receptors (**d**) as a function of time. Source data are provided as a Source Data file.

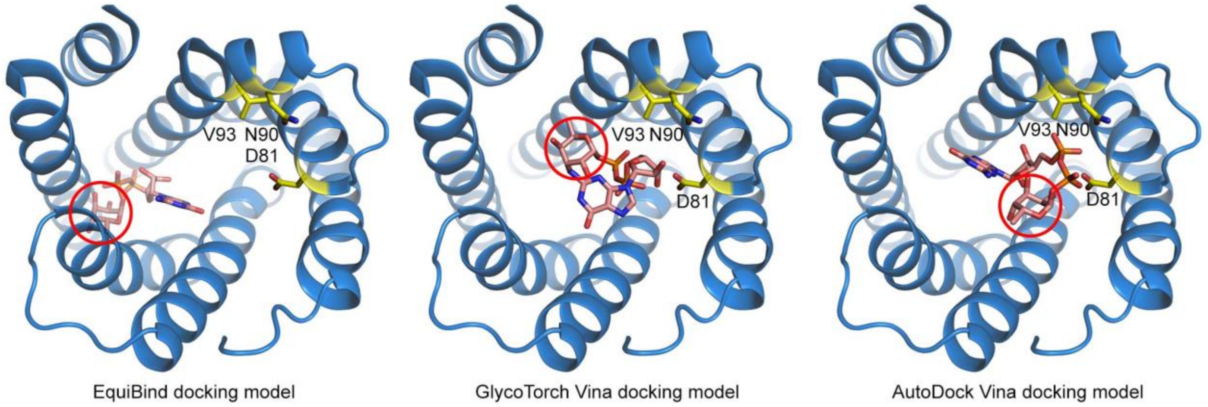

Supplementary Fig. 8 | The top-one ranked docking models of GDP-Fuc to the AlphaFold2-predicted human P2Y14 structure by different methods. Ligands and key residues are shown as sticks. Source data are provided in the simulation files.

Supplementary Tables

Supplementary Table 1 | Five-fold cross validation of DeepGlycanSite on the independent dataset T145.

Fold	MCC	Precision	Balanced Accuracy
1	0.609 ± 0.276	0.592 ± 0.281	0.833 ± 0.153
2	0.603 ± 0.288	0.600 ± 0.300	0.823 ± 0.156
3	0.591 ± 0.284	0.621 ± 0.303	0.802 ± 0.156
4	0.625 ± 0.292	0.631 ± 0.306	0.829 ± 0.156
5	0.596 ± 0.304	0.623 ± 0.322	0.807 ± 0.165
Average	0.605 ± 0.012	0.613 ± 0.015	0.819 ± 0.012

Data represent means ± standard deviation. Source data are provided as a Source Data file.

Supplementary Table 2 | Comparing DeepGlycanSite with previous methods in predicting 32 monosaccharide-binding sites of the independent dataset T145.

Method	MCC	Precision	Balanced accuracy
StackCBPred	0.020 ± 0.100***	0.042 ± 0.031***	0.532 ± 0.126***
Fpocket	0.116 ± 0.276***	0.122 ± 0.215***	0.575 ± 0.177***
SiteMap	0.205 ± 0.360***	0.167 ± 0.203***	0.709 ± 0.226***
DeepPocket	0.061 ± 0.556***	0.166 ± 0.156***	0.710 ± 0.212***
PeSTo	0.296 ± 0.289***	0.172 ± 0.120***	0.837 ± 0.162**
DeepGlycanSite	0.687 ± 0.266	0.622 ± 0.278	0.900 ± 0.146

Data represent means ± standard deviation. Two-tailed Mann-Whitney U test is used to determine statistical difference between DeepGlycanSite and an alternative method. ** indicates P is less than 0.01, *** indicates P is less than 0.001. From top to bottom and left to right, the P values of the significantly different groups are 4.9E-10, 6.5E-8, 3.2E-7, 2.0E-7, 2.3E-7, 1.9E-9, 1.7E-8, 8.4E-8, 2.4E-8, 1.7E-8, 1.2E-9, 4.6E-8, 5.1E-5, 1.9E-4 and 2.9E-3. Source data are provided as a Source Data file.

Supplementary Table 3 | Comparing DeepGlycanSite with previous methods in predicting 13 disaccharide-binding sites of the independent dataset T145.

Method	MCC	Precision	Balanced accuracy
StackCBPred	0.070 ± 0.063***	0.056 ± 0.044***	0.578 ± 0.069***
Fpocket	0.242 ± 0.347**	0.224 ± 0.269**	0.650 ± 0.220
SiteMap	0.162 ± 0.464**	0.200 ± 0.238***	0.663 ± 0.220
DeepPocket	0.306 ± 0.496*	0.294 ± 0.239**	0.770 ± 0.231
PeSTo	0.343 ± 0.149**	0.172 ± 0.099***	0.864 ± 0.126
DeepGlycanSite	0.677 ± 0.278	0.709 ± 0.308	0.836 ± 0.133

Data represent means ± standard deviation. Two-tailed Mann-Whitney U test is used to determine statistical difference between DeepGlycanSite and an alternative method. * indicates P is less than 0.05, ** indicates P is less than 0.01, *** indicates P is less than 0.001. From top to bottom and left to right, the P values of the significantly different groups are 2.2E-4, 5.6E-3, 2.7E-3, 4.0E-2, 2.3E-3, 2.7E-4, 1.1E-3, 6.0E-4, 3.1E-3, 5.8E-4 and 2.2E-4. Source data are provided as a Source Data file.

Supplementary Table 4 | Comparing DeepGlycanSite with previous methods in predicting 20 oligosaccharide-binding sites of the independent dataset T145.

Method	MCC	Precision	Balanced accuracy
StackCBPred	0.027 ± 0.063***	0.059 ± 0.035***	0.526 ± 0.079***
Fpocket	0.142 ± 0.293**	0.176 ± 0.278***	0.581 ± 0.167**
SiteMap	0.130 ± 0.397***	0.173 ± 0.229***	0.619 ± 0.199**
DeepPocket	0.410 ± 0.448	0.400 ± 0.267**	0.789 ± 0.211
PeSTo	0.397 ± 0.195**	0.275 ± 0.146***	0.820 ± 0.144
DeepGlycanSite	0.612 ± 0.261	0.658 ± 0.274	0.810 ± 0.135

Data represent means ± standard deviation. Two-tailed Mann-Whitney U test is used to determine statistical difference between DeepGlycanSite and an alternative method. ** indicates P is less than 0.01, *** indicates P is less than 0.001. From top to bottom and left to right, the P values of the significantly different groups 1.4E-6, 9.3E-5, 1.8E-4, 4.7E-3, 1.1E-6, 5.5E-5, 1.5E-5, 5.7E-3, 5.2E-5, 7.1E-6, 2.5E-4 and 4.3E-3. Source data are provided as a Source Data file.

Supplementary Table 5 | Comparing DeepGlycanSite with previous methods in predicting 69 nucleotide-binding sites of the independent dataset T145.

Method	MCC	Precision	Balanced accuracy
StackCBPred	-0.004 ± 0.086***	0.055 ± 0.032***	0.500 ± 0.093***
Fpocket	0.246 ± 0.343***	0.243 ± 0.301***	0.650 ± 0.203***
SiteMap	0.303 ± 0.414***	0.248 ± 0.194***	0.784 ± 0.203
DeepPocket	0.382 ± 0.432***	0.343 ± 0.231***	0.802 ± 0.191
PeSTo	0.397 ± 0.267***	0.288 ± 0.179***	0.824 ± 0.155
DeepGlycanSite	0.655 ± 0.258	0.671 ± 0.279	0.836 ± 0.136

Data represent means ± standard deviation. Two-tailed Mann-Whitney U test is used to determine statistical difference between DeepGlycanSite and an alternative method. *** indicates P is less than 0.001. From top to bottom and left to right, the P values of the significantly different groups 6.9E-21, 5.6E-10, 3.2E-10, 1.5E-6, 1.1E-9, 5.1E-19, 1.2E-11, 8.9E-15, 6.8E-11, 1.7E-13, 2.2E-20 and 2.2E-6. Source data are provided as a Source Data file.

Supplementary Table 6 | Comparing DeepGlycanSite with previous methods in predicting 8 glycolipid-binding sites of the independent dataset T145.

Method	MCC	Precision	Balanced accuracy
StackCBPred	0.045 ± 0.033*	0.025 ± 0.010	0.568 ± 0.055
Fpocket	-0.030 ± 0.014	0.000 ± 0.000	0.477 ± 0.014*
SiteMap	-0.002 ± 0.081	0.013 ± 0.034	0.506 ± 0.091
DeepPocket	0.015 ± 0.096	0.025 ± 0.044	0.513 ± 0.104
PeSTo	-0.210 ± 0.463	0.040 ± 0.053	0.532 ± 0.078
DeepGlycanSite	0.050 ± 0.173	0.078 ± 0.184	0.523 ± 0.080

Data represent means ± standard deviation. Two-tailed Mann-Whitney U test is used to determine statistical difference between DeepGlycanSite and an alternative method. * indicates P is less than 0.05, ** indicates P is less than 0.01. From top to bottom and left to right, the P values of the significantly different groups are 5.0E-2 and 3.5E-2. Source data are provided as a Source Data file.

Supplementary Table 7 | Comparing DeepGlycanSite with two re-implemented machine learning models on the independent dataset T145.

Method	MCC	Precision	Balanced accuracy
SVM	0.068 ± 0.544***	0.408 ± 0.353***	0.597 ± 0.098***
XGBoost	0.126 ± 0.470***	0.429 ± 0.361***	0.588 ± 0.095***
DeepGlycanSite	0.625 ± 0.292	0.631 ± 0.306	0.829 ± 0.156

Data represent means ± standard deviation. Two-tailed Mann-Whitney U test is used to determine statistical difference between DeepGlycanSite and an alternative method. *** indicates P is less than 0.001. From top to bottom and left to right, the P values of the significantly different groups are 1.4E-22, 2.2E-22, 3.9E-6, 1.5E-6, 1.4E-26 and 7.8E-27. Source data are provided as a Source Data file.

Supplementary Table 8 | Comparing DeepGlycanSite with previous methods on the independent dataset TM29.

Method	MCC	Precision	Balanced accuracy
StackCBPred	0.026 ± 0.104***	0.069 ± 0.041***	0.529 ± 0.103***
Fpocket	0.212 ± 0.304***	0.207 ± 0.233***	0.641± 0.198***
SiteMap	0.145 ± 0.466 ***	0.189 ± 0.176***	0.695 ± 0.199**
DeepPocket	0.356 ± 0.357***	0.342 ± 0.217***	0.756 ± 0.170
PeSTo	0.324 ± 0.190***	0.182 ± 0.149***	0.823 ± 0.163
DeepGlycanSite	0.688 ± 0.233	0.755 ± 0.228	0.839 ± 0.136

Data represent means ± standard deviation. Two-tailed Mann-Whitney U test is used to determine statistical difference between DeepGlycanSite and an alternative method. ** indicates P is less than 0.01, *** indicates P is less than 0.001. From top to bottom and left to right, the P values of the significantly different groups are 8.1E-10, 2.1E-6, 2.0E-7, 1.5E-5, 2.7E-7, 1.2E-9, 2.0E-8, 3.0E-9, 1.3E-7, 4.5E-9, 6.6E-9, 7.4E-4 and 6.5E-3. Source data are provided as a Source Data file.

Supplementary Table 9 | Comparing DeepGlycanSite with previous methods on the independent dataset TS116.

Method	MCC	Precision	Balanced accuracy
StackCBPred	0.016 ± 0.082***	0.047 ± 0.031***	0.524 ± 0.099***
Fpocket	0.186 ± 0.329***	0.191 ± 0.288***	0.611 ± 0.196***
SiteMap	0.247 ± 0.379***	0.204 ± 0.215***	0.722 ± 0.224***
DeepPocket	0.271 ± 0.503***	0.279 ± 0.242***	0.761 ± 0.218
PeSTo	0.340 ± 0.324***	0.249 ± 0.168***	0.814 ± 0.165
DeepGlycanSite	0.609 ± 0.303	0.600 ± 0.315	0.827 ± 0.161

Data represent means ± standard deviation. Two-tailed Mann-Whitney U test is used to determine statistical difference between DeepGlycanSite and an alternative method. *** indicates P is less than 0.001. From top to bottom and left to right the P values of the significantly different groups are 1.4E-27, 5.7E-17, 9.3E-15, 6.1E-10, 1.7E-12, 3.4E-24, 2.5E-17, 1.7E-18, 5.5E-14, 1.0E-16, 1.1E-26, 5.7E-14 and 7.9E-4. Source data are provided as a Source Data file.

Supplementary Table 10 | Comparing DeepGlycanSite with alternative methods on the independent dataset T59.

Method	MCC	Precision	Balanced accuracy
StackCBPred	0.017 ± 0.085***	0.050 ± 0.034***	0.523 ± 0.093***
Fpocket	0.190 ± 0.324***	0.195 ± 0.281***	0.617 ± 0.198***
SiteMap	0.169 ± 0.452***	0.187 ± 0.199***	0.698 ± 0.214
DeepPocket	0.287 ± 0.476***	0.288 ± 0.227***	0.760 ± 0.207
PeSTo	0.258 ± 0.349***	0.204 ± 0.148***	0.769 ± 0.172
DeepGlycanSite	0.532 ± 0.299	0.549 ± 0.302	0.778 ± 0.161

Data represent means ± standard deviation. Two-tailed Mann-Whitney U test is used to determine statistical difference between DeepGlycanSite and an alternative method. *** indicates P is less than 0.001. From top to bottom and left to right, the P values of the significantly different groups are 1.1E-12, 5.8E-7, 4.0E-7, 6.1E-4, 7.2E-7, 1.2E-10, 9.0E-8, 1.5E-9, 1.0E-6, 5.2E-9, 3.6E-12 and 7.4E-5. Source data are provided as a Source Data file.

Supplementary Table 11 | Comparing DeepGlycanSite with alternative methods on the independent dataset $T59_{AF2}$.

Method	MCC	Precision	Balanced Accuracy
Fpocket	-0.048 ± 0.476***	0.135 ± 0.189***	0.571 ± 0.123***
SiteMap	0.233 ± 0.241***	0.172 ± 0.170***	0.690 ± 0.189*
DeepPocket	-0.488 ± 0.669***	0.131 ± 0.230***	0.590 ± 0.154***
PeSTo	0.289 ± 0.270***	0.220 ± 0.165***	0.762 ± 0.161
DeepGlycanSite	0.467 ± 0.272	0.436 ± 0.274	0.777 ± 0.153

Data represent means ± standard deviation. Two-tailed Mann-Whitney U test is used to determine statistical difference between DeepGlycanSite and an alternative method. * indicates P is less than 0.05, *** indicates P is less than 0.001. From top to bottom and left to right the P values of the significantly different groups are 2.4E-10, 2.0E-6, 2.6E-11, 1.7E-6, 5.6E-9, 2.6E-7, 1.6E-9, 1.3E-5, 1.3E-9, 1.2E-2 and 9.2E-7. Source data are provided as a Source Data file.

Supplementary Table 12 | Ablation results of DeepGlycanSite on the independent dataset T145.

ID	Ablated item	MCC	Precision	Balanced accuracy
1	Geometric features	0.608 ± 0.269	0.569 ± 0.276*	**0.845 ± 0.148**
2	Evolutionary features	0.559 ± 0.279**	0.532 ± 0.287**	0.816 ± 0.157
3	Scalar-vector interactions in the updating units	0.420 ± 0.246***	0.399 ± 0.234***	0.746 ± 0.145***
4	Transformer	0.570 ± 0.331	0.567 ± 0.294*	0.840 ± 0.158
5	None	0.625 ± 0.292	0.631 ± 0.306	0.829 ± 0.156

Data represent means ± standard deviation. Two-tailed Mann-Whitney U test is used to determine statistical difference between DeepGlycanSite and ablated DeepGlycanSite. * indicates P is less than 0.05, ** indicates P is less than 0.01, *** indicates P is less than 0.001. From top to bottom and left to right, the P values of the significantly different groups are 9.5E-3, 1.3E-11, 2.2E-2, 1.4E-3, 4.0E-12, 3.2E-2 and 5.3E-7. Source data are provided as a Source Data file.

Supplementary Table 13 | Comparing DeepGlycanSite$_{+Ligand}$ with previous docking methods on the independent dataset TM175.

Method	MCC	Precision	Balanced accuracy
EquiBind	0.278 ± 0.260***	0.320 ± 0.287***	0.635 ± 0.130***
GlycoTorch Vina	0.340 ± 0.392***	0.351 ± 0.380***	0.677 ± 0.202***
AutoDock Vina	0.344 ± 0.390***	0.355 ± 0.381***	0.679 ± 0.199***
DiffDock	0.367 ± 0.358***	0.402 ± 0.368***	0.678 ± 0.175***
DeepGlycanSite$_{+Ligand}$	0.538 ± 0.321	0.504 ± 0.327	0.806 ± 0.163

Data represent means ± standard deviation. Two-tailed Mann-Whitney U test is used to determine statistical difference between DeepGlycanSite$_{+Ligand}$ and an alternative method. *** indicates *P* is less than 0.001. From top to bottom and left to right, the P values of the significantly different groups are 2.4E-13, 3.1E-6, 3.4E-6, 1.2E-5, 8.8E-8, 1.2E-5, 2.4E-5, 2.8E-3, 4.0E-20, 1.4E-8, 1.2E-8 and 9.4E-11. Source data are provided as a Source Data file.

Supplementary Table 14 | Five-fold cross validation of DeepGlycanSite$_{+Ligand}$ on the independent dataset TM175.

Fold	MCC	Precision	Balanced Accuracy
1	0.527 ± 0.325	0.498 ± 0.326	0.795 ± 0.167
2	0.538 ± 0.321	0.504 ± 0.327	0.806 ± 0.163
3	0.536 ± 0.326	0.552 ± 0.344	0.775 ± 0.161
4	0.534 ± 0.323	0.493 ± 0.321	0.804 ± 0.169
5	0.530 ± 0.325	0.518 ± 0.334	0.787 ± 0.165
Average	0.533 ± 0.004	0.513 ± 0.023	0.793 ± 0.012

Data represent means ± standard deviation. Source data are provided as a Source Data file.

Supplementary Table 15 | Ablation results of DeepGlycanSite$_{+Ligand}$ on the independent dataset TM175.

ID	Ablated item	MCC	Precision	Balanced accuracy
1	Molecule features	0.500 ± 0.322	0.482 ± 0.318	0.777 ± 0.177
2	Ligand vector	0.415 ± 0.310***	0.390 ± 0.291***	0.744 ± 0.184**
3	Ligand graph	0.495 ± 0.313	0.503 ± 0.307	0.797 ± 0.174*
4	All ligand information	0.399 ± 0.324***	0.380 ± 0.318***	0.731 ± 0.182***
5	None	0.538 ± 0.321	0.504 ± 0.327	0.806 ± 0.163

Data represent means ± standard deviation. Two-tailed Mann-Whitney U test is used to determine statistical difference between DeepGlycanSite$_{+Ligand}$ and ablated DeepGlycanSite$_{+Ligand}$. * indicates P is less than 0.05, ** indicates P is less than 0.01, *** indicates P is less than 0.001. From top to bottom and left to right, the P values of the significantly different groups are 4.0E-6, 6.3E-5, 7.6E-6, 2.5E-6, 2.4E-3 and 9.1E-5. Source data are provided as a Source Data file.

Supplementary Table 16 | The EC_{50} values for P2Y14 and its mutant activation upon GDP-Fuc binding in the calcium mobilization assays.

Construct	EC_{50} (μM)	*n*	Statistics	Comment
WT	0.49 ± 0.04	12	T.TEST	
L79A	0.54 ± 0.14	3	NS	WT vs. L79A
G80A	0.94 ± 0.11	4	P = 2.1E-4	WT vs. G80A
G80R	3.31 ± 0.27	6	P = 1.4E-10	WT vs. G80R
D81A	5.22 ± 0.49	6	P = 2.3E-10	WT vs. D81A
N90A	1.02 ± 0.03	3	P = 1.4E-5	WT vs. N90A
V91A	0.61 ± 0.15	3	NS	WT vs. V91A
V91R	0.62 ± 0.19	3	NS	WT vs. V91R
F92M	0.58 ± 0.09	3	NS	WT vs. F92M
V93A	1.39 ± 0.13	6	P = 1.9E-7	WT vs. V93A
V93M	4.13 ± 0.58	5	P = 4.2E-8	WT vs. V93M
Q169A	0.51 ± 0.04	5	NS	WT vs. Q169A
I170A	0.85 ± 0.07	5	P = 1.8E-4	WT vs. I170A
A285G	0.45 ± 0.04	5	NS	WT vs. A285G
A286G	0.48 ± 0.03	3	NS	WT vs. A286G
A286L	0.45 ± 0.05	3	NS	WT vs. A286L
N287A	Not detectable	3	-	WT vs. N287A

NS indicates the difference is not statistically significant. The t test is two-sided. Source data are provided as a Source Data file.

Supplementary Table 17 | The EC_{50} values for P2Y14 and its mutant activation upon GDP binding in the calcium mobilization assays.

Construct	EC_{50} (μM)	*n*	Statistics	Comment
WT	0.24 ± 0.01	10	T.TEST	
L79A	0.26 ± 0.06	3	NS	WT vs. L79A
G80A	0.35 ± 0.02	3	P = 5.0E-4	WT vs. G80A
G80R	0.10 ± 0.01	6	P = 4.0E-7	WT vs. G80R
D81A	0.17 ± 0.01	6	P = 2.1E-4	WT vs. D81A
N90A	0.22 ± 0.03	3	NS	WT vs. N90A
V91A	0.24 ± 0.02	3	NS	WT vs. V91A
V91R	0.23 ± 0.03	3	NS	WT vs. V91R
F92M	0.27 ± 0.04	3	NS	WT vs. F92M
V93A	0.41 ±0.06	3	P = 4.1E-4	WT vs. V93A
V93M	0.40 ± 0.02	6	P = 4.6E-6	WT vs. V93M
Q169A	0.27 ± 0.00	3	NS	WT vs. Q169A
I170A	0.31 ± 0.02	3	P = 1.3E-2	WT vs. I170A
A285G	0.20 ± 0.03	3	NS	WT vs. A285G
A286G	0.26 ±0.01	3	NS	WT vs. A286G
A286L	0.26 ± 0.04	3	NS	WT vs. A286L
N287A	Not detectable	3	-	WT vs. N287A

NS indicates the difference is not statistically significant. The t-test is two-sided. Source data are provided as a Source Data file.

Supplementary Table 18 | Node features for protein graph construction.

Features	Size	Description
Residue type	20	One hot encoding for residue type ALA, ARG, ASN, ASP, CYS, GLN, GLU, GLY, HIS, ILE, LEU, LYS, MET, PHE, PRO, SER, THR, TRP, TYR, and VAL
Intra-residual distance	5	The maximum and minimum atomic distances within a residue and the distances of three backbone atom pairs (Cα-O, O-N and N-C) in unit Å (all multiplied by 0.1)
Intra-residual dihedral	4	Three backbone dihedral angles (φ, ψ and ω) and a sidechain dihedral angle (χ_1) are calculated for the current residue. φ is the dihedral angle determined by C_{n-1}, N_n, $C\alpha_n$ and C_n, ψ is the dihedral angle determined by N_n, $C\alpha_n$, C_n and N_{n+1}, ω is the dihedral angle determined by $C\alpha_n$, C_n, N_{n+1} and $C\alpha_{n+1}$, and χ_1 is the dihedral angle determined by N_n, $C\alpha_n$, $C\beta_n$ and $C\gamma_n$ (all multiplied by 0.01)
Evolutional features	1280	ESM-2 (esm2_t33_650M_UR50D) feature on residue level, each chain was separately used as input

Supplementary Table 19 | Edge features for protein graph construction.

Features	Size	Description
Connectivity	1	One-hot encoding for the adjacency of two residues in the sequence
Inter-residual distance	3	Cα-Cα distance, Cβ-Cβ distance and distance between sidechain center of mass of the two residues (all multiplied by 0.1)
Inter-residual dihedral	5	θ_0 measures rotation along the virtual axis connecting the Cβ atoms of residue i and residue j. θ_1-θ_4 specify the direction of the Cβ atom of residue j (i) in a reference frame centered on residue i (j)<br>θ_0 is determined by $C\alpha_i$, $C\beta_i$, $C\beta_j$, and $C\alpha_j$;<br>θ_1 is determined by N_i, $C\alpha_i$, $C\beta_i$, and $C\beta_j$;<br>θ_2 is determined by $C\beta_i$, $C\beta_j$, $C\alpha_j$, and N_j;<br>θ_3 is determined by N_j, $C\alpha_j$, $C\beta_j$, and $C\beta_i$;<br>θ_4 is determined by $C\beta_j$, $C\beta_i$, $C\alpha_i$, and N_i (all multiplied by 0.01)

Supplementary Table 20 | Node features for carbohydrate graph construction.

Features	Size	Description
Atom type	17	One hot encoding for the following atom types: C, N, O, S, F, P, Cl, Br, I, B, Si, Fe, Zn, Cu, Mn, Mo, and others
Degrees	7	One hot encoding for atom degrees 0-6
Hybridization type	6	One hot encoding for the following types: SP, SP2, SP3, SP3D, SP3D2, and others
Number of hydrogens	5	One hot encoding for the total number of hydrogens binding on this atom (from 0 to 4)
Chiral property	3	A 3-D one-hot vector to describe R, S or an atom with unknown chiral property. If the atom has no chiral property, the vector is all-zero
Formal charge	1	Formal charge defined by Rdkit
Number of radical electrons	1	Number of radical electrons defined by Rdkit
Aromaticity	1	One hot encoding for aromatic atom or not

Supplementary Table 21 | Edge features for carbohydrate graph construction.

Features	Size	Description
Bond type	4	One hot encoding for the following bond types: single, double, triple and aromatic bonds
Conjugation	1	One hot encoding for conjugated bond or not
Bond in ring	1	One hot encoding for bond in a ring or not

Supplementary Table 22 | Hyperparameters for DeepGlycanSite models.

Hyperparameters	DeepGlycanSite	$\text{DeepGlycanSite}_{+\text{Ligand}}$
Batch size	3	4
Random seed	1	0
Dropout rate	0.29	0.15
Hidden dimension size	512	256
Radial basis function number	128	64
l_{max} of spherical harmonics	1	2
ViSNet attention layer heads	32	16
ViSNet attention layers	11	11
Transformer attention layer heads	16	8
Transformer encoder layers	5	7
Transformer decoder layers	7	7
Learning rate	0.99E-5	2.75E-5
Weight decay	1.85E-5	4.02E-5
ReduceLROnPlateau learning rate factor	0.71	0.79
ReduceLROnPlateau patience	10	4
Minimum learning rate	3.48E-8	1.65E-8
Loss α	0.23	0.32
Loss γ	3.79	3.29

Supplementary Table 23 | System setup for MD simulations.

Setup item	GDP-bound receptor	GDP-Fuc-bound receptor
Simulation box dimensions	60.4×60.4×135.7 $Å^3$	60.2×60.2×135.7 $Å^3$
Total atom number	45,495	45,248
Total water number	10,084	10,039
Lipid (POPC) number	71	70
Salt concentration	0.15 mol/L	0.15 mol/L

Supplementary Methods

Model architecture

An exponentially modified Gaussian radial distribution function, namely equation (S1) was applied for distance normalization.

$$g(\vec{\boldsymbol{r}}_{ij}) = \phi(\|\vec{\boldsymbol{r}}_{ij}\|) \cdot \exp(-\beta_k(\exp(-\|\vec{\boldsymbol{r}}_{ij}\|) - \mu_k)^2) \tag{S1}$$

Where β_k and μ_k are optional learnable parameters that specify the center and width of the function. $\phi(\cdot)$ is a cosine cutoff function.

Neighborhood embedding is defined as equation (S2)

$$h_{ni} = \sum_{j \in N(i)} W_{n1}(f_{nj}) \odot W_{e1}(g(\vec{\boldsymbol{r}}_{ij})) \tag{S2}$$

Where W_{n1} and W_{e1} denote weight for neighborhood embedding of node and edge features, respectively. $\odot$ is the Hadamard product.

Node and edge vectors were initialized as equation (S3-S4).

$$\vec{\boldsymbol{v}}_n = [0,0,\ldots,0] \in \mathbb{R}^{(l_{max}+1)^2-1} \tag{S3}$$

$$\vec{\boldsymbol{v}}_e = Sphere((pos_i - pos_j) \,/\, \|(pos_i - pos_j)\|_2)) \tag{S4}$$

Namely node vector was initialized as an all-zero vector according to l_{max} for spherical harmonics function ($Sphere$) and the edge vector was initialized as the normalized edge distance with spherical harmonics.

To calculate intermediate scalar and vector m_{ij}^l and $\vec{\boldsymbol{m}}_{ij}^l$ in Scalar2Vec module, equations (S5-S8) were applied.

$$\alpha_{ij}^l = \sigma\big((W_Q^l h_i^l) \cdot (W_K^l(h_j^l) \odot Dense_K^l(e_{ij}^l))^T\big) \tag{S5}$$

$$m_{ij}^l = \alpha_{ij}^l \cdot \phi(\|\vec{\boldsymbol{r}}_{ij}\|) \cdot (W_V^l(h_j^l) \odot Dense_V^l(e_{ij}^l)) \tag{S6}$$

$$\vec{\boldsymbol{m}}_{ij}^l = (Dense_u^l(m_{ij}^l) \odot \vec{\boldsymbol{v}}_e) + (Dense_v^l(m_{ij}^l) \odot \vec{\boldsymbol{v}}_j^l) \tag{S7}$$

$$m_i^l = \sum_{j \in N(i)} m_{ij}^l, \vec{\boldsymbol{m}}_i^l = \sum_{j \in N(i)} \vec{\boldsymbol{m}}_{ij}^l \tag{S8}$$

Where α_{ij}^l is the attention coefficient for message passing between node i and j, l is the index of attention layer, σ denotes the sigmoid linear unit activation function, W_Q, W_K, and W_V are learnable weight matrices for query, key and value in attention, and $Dense$ refers to one learnable weight matrix with a sigmoid linear unit activation function.

The gated equivariant block is defined in equation (S9).

$$n_i^l = W_3^l(Dense_2^l([\|W_1^l \vec{\boldsymbol{v}}_i^l\|, n_i^l])) \tag{S9}$$

Where brackets mean concatenation.

The transformer structure is described in equations (S10-S14).

$$Q = W_Q n_i^l, K = W_K n_i^l, V = W_V n_i^l \tag{S10}$$

$$Attention(Q, K, V) = softmax(\frac{QK^T}{\sqrt{d_{node}}})V \tag{S11}$$

$$head_i = Attention(QW_i^Q, KW_i^K, VW_i^V) \tag{S12}$$

$$MultiHead(Q,K,V) = [head_1, \dots, head_n]W^O \tag{S13}$$

$$n_i^{l+1} = W^{O1} MultiHead(Q,K,V) \tag{S14}$$

Where d_{node} means the dimension of node embeddings. Such layers were overlapped, and all nodes were connected during the self-attention process.

In DeepGlycanSite$_{\text{+Ligand}}$, linear layers for feature projecting were defined in equations (S15-S16).

$$n_i^0 = W_n^0(n_i) + b_n^0 \tag{S15}$$

$$e_{ij}^0 = W_{ij}^0(e_{ij}) + b_{ij}^0 \tag{S16}$$

Where b_n^0 and b_{ij}^0 mean the bias parameters for node and edge, respectively.

MetaConv layers facilitated the integration of neighboring atom features with the central atom, as demonstrated by equations (S17-S18).

$$n_i^{l+1} = MLP([n_i^l, \frac{1}{\|j\|} \sum_j MLP([n_i^l, e_{ij}^{l+1}])]) \tag{S17}$$

$$e_{ij}^{l+1} = MLP([n_i^l, n_j^l, e_{ij}^l]) \tag{S18}$$

Where l is the index of the Metaconv layer, MLP represents multi-layer perceptron. ResBlock layers were described in equations (S19-S22).

$$n_i^{l1} = MLP_{ds}(n_i^l),\ e_{ij}^{l1} = MLP_{ds}(e_{ij}^l) \quad \text{(S19)}$$

$$n_i^{l2}, e_{ij}^{l2} = Metaconv(n_i^{l1}, n_j^{l1}, e_{ij}^{l1}) \quad \text{(S20)}$$

$$n_i^{l3} = MLP_{up}(n_i^{l2}),\ e_{ij}^{l3} = MLP_{up}(e_{ij}^{l2}) \quad \text{(S21)}$$

$$n_i^{l+1} = ELU(n_i^l + n_i^{l3}),\ e_{ij}^{l+1} = ELU(e_{ij}^l + e_{ij}^{l3}) \quad \text{(S22)}$$

The term "ds" means down-sampling and the MLP_{ds} projects the initial vector to half of its original dimensions. MLP_{up} projects the initial vector to twice its dimensions. To extract the graph-level features, a 'set2set' operation described in equations (S23-S25) was utilized.

$$q_t = LSTM(q_{t-1}) \quad \text{(S23)}$$

$$\alpha_{i,t} = softmax(n_i \cdot q_t) \quad \text{(S24)}$$

$$q_t^* = q_t \| \sum_{i=1}^{N} \alpha_{i,t} n_i \quad \text{(S25)}$$

Where q is the query vector. LSTM is short for the Long Short Term Memory network.

Ablation experiments

In the ablation studies for DeepGlycanSite, several modifications were made to analyze the impact of different features and network components. Geometric features such as distance and dihedral were removed from the nodes and edges, reducing node feature dimensions to 1300 and the edge dimension to 1. Evolutionary features were also adjusted by removing the 1280

dimensions representing the ESM-2 pretraining information. To evaluate the effect of scalar-vector interactions, ViSNet was replaced with SchNet in the updating units. Moreover, the transformer was replaced with an MLP applied directly to each node following the equivalent gate.

In the DeepGlycanSite$_{+\text{ligand}}$ model, the first three modifications included removing the concatenation of ligand graph features with molecule features, using the original ViSNet for updates without involving the ligand vector, and allowing only molecule features into the ligand vector while the transformer processed outputs from ReceptorNet exclusively. To remove all ligand information, the network structure was kept the same as in DeepGlycanSite, but labeling followed the DeepGlycanSite$_{+\text{ligand}}$ criteria, highlighting a potential area of confusion as a protein can bind multiple ligands; DeepGlycanSite labels both ligand sites as positive, whereas DeepGlycanSite$_{+\text{ligand}}$ only labels one.

All ablation studies utilized the same division of training and validation sets as the original training for both DeepGlycanSite and DeepGlycanSite$_{+\text{ligand}}$.

Re-implement of machine learning models

In re-implement of traditional machine learning techniques for DeepGlycanSite data, we extracted feature vectors with 1309 dimensions from our training datasets. The labeling of each amino acid residue was consistent with its classification in DeepGlycanSite, and to preserve the integrity of protein data, residues from the same protein were allocated to the same batch

for training purposes. For the support vector machine (SVM) model, we employed the scikit-learn library, configured with a hinge loss function. In the case of the eXtreme Gradient Boosting (XGBoost) model, we used the XGBoost Python package, setting the hyperparameters to include a maximum depth of 6, a learning rate of 0.3, with the objective set to 'binary:logistic' and the evaluation metric to 'logloss'.

Molecule docking

In AutoDock Vina[1] and GlycoTorch Vina[2], ligands were converted from PDB format to PDBQT format using MGLTools' prepare_ligand4.py[3]. Protein preparation was performed using Schrödinger's Protein Preparation Wizard in Maestro. Subsequently, proteins were converted to PDBQT files using MGLTools' prepare_receptor4.py[3]. The search space was defined by a box centered on the average value of all atom coordinates, with x, y, and z lengths extending 5 Å beyond the largest coordinate values. The grid spacing was set to default 0.375 Å, and the docking exhaustiveness was set to 12. In Diffdock[4], ligands were firstly converted from PDB format to SDF files using Open Babel[5]. atom connections that did not obey RDKit sanitization rules were manually adjusted for Diffdock ligand input. At last, the protein and ligand were input separately, and the docking pose with the highest confidence score was selected as the final result. For Equibind[6], ligands were also converted from PDB format to SDF files using Open Babel[5].

Evaluation metrics

The MCC is defined as the equation (S26):

$$MCC = \frac{(TP \cdot TN - FP \cdot FN)}{\sqrt{(TP+FP) \cdot (TP+FN) \cdot (TN+FP) \cdot (TN+FN)}} \quad \text{(S26)}$$

MCC ranges from -1 to 1; a small value of -1 indicates that no carbohydrate-binding residue is correctly predicted, and a large value of 1 indicates that all carbohydrate-binding residues are correctly predicted.

The precision is defined as equation (S27):

$$Precision = \frac{TP}{TP+FP} \quad \text{(S27)}$$

The balanced accuracy (BACC) is defined as equation (S28):

$$Balanced\ accuracy = \frac{1}{2}\left(\frac{TP}{TP+FN} + \frac{TN}{TN+FP}\right) \quad \text{(S28)}$$

Supplementary References

1. Trott O, Olson AJ. AutoDock Vina: improving the speed and accuracy of docking with a new scoring function, efficient optimization, and multithreading. *J. Comput. Chem.* **31**, 455-461 (2010).
2. Boittier ED, Burns JM, Gandhi NS, Ferro V. GlycoTorch Vina: Docking Designed and Tested for Glycosaminoglycans. *J. Chem. Inf. Model.* **60**, 6328-6343 (2020).
3. Morris GM, et al. AutoDock4 and AutoDockTools4: Automated docking with selective receptor flexibility. *J. Comput. Chem.* **30**, 2785-2791 (2009).
4. Corso G, Stärk H, Jing B, Barzilay R, Jaakkola T. Diffdock: Diffusion steps, twists, and turns for molecular docking. In *International Conference on Learning Representations* (eds Kigali, Rwanda) (Ithaca, NY. 2023).
5. O'Boyle NM, Banck M, James CA, Morley C, Vandermeersch T, Hutchison GR. Open Babel: An open chemical toolbox. *J. Cheminfo.* **3**, 33 (2011).
6. Stark H, Ganea OE, Pattanaik L, Barzilay R, Jaakkola T. EQUIBIND: Geometric Deep Learning for Drug Binding Structure Prediction. In *Proceedings of Machine Learning Research* (eds Baltimore, MD, USA) (ACM. 2022).
7. Vanommeslaeghe K, et al. CHARMM general force field: A force field for drug-like molecules compatible with the CHARMM all-atom additive biological force fields. *J. Comput. Chem.* **31**, 671-690 (2010).
8. Huang J, et al. CHARMM36m: an improved force field for folded and intrinsically disordered proteins. *Nat. Methods* **14**, 71-73 (2017).
9. MacKerell AD, et al. All-Atom Empirical Potential for Molecular Modeling and Dynamics Studies of Proteins. *J. Phys. Chem. B* **102**, 3586-3616 (1998).
10. Salomon-Ferrer R, Götz AW, Poole D, Le Grand S, Walker RC. Routine Microsecond Molecular Dynamics Simulations with AMBER on GPUs. 2. Explicit Solvent Particle Mesh Ewald. *J. Chem. Theory. Comput.* **9**, 3878-3888 (2013).
11. Evans DJ, Holian BL. The Nose–Hoover thermostat. *J. Chem. Phys.* **83**, 4069-4074 (1985).
12. Parrinello M, Rahman A. Polymorphic transitions in single crystals: A new molecular dynamics method. *J. Appl. Phys.* **52**, 7182-7190 (1981).
13. Ryckaert J-P, Ciccotti G, Berendsen HJC. Numerical integration of the cartesian equations of motion of a system with constraints: molecular dynamics of n-alkanes. *J. Comput. Phys.* **23**, 327-341 (1977).
14. Darden T, York D, Pedersen L. Particle mesh Ewald: An N · log(N) method for Ewald sums in large systems. *J. Chem. Phys.* **98**, 10089-10092 (1993).
