# Supplementary material for: Highly accurate carbohydrate-binding site prediction with DeepGlycanSite
Source: Nat Commun. 2024 Jun 17;15:5163. doi: 10.1038/s41467-024-49516-2 (PMC11183243; doi:10.1038/s41467-024-49516-2)
Supplement: Supplementary file 2 — Reporting Summary [file 41467_2024_49516_MOESM2_ESM.pdf]

nature portfolio

Corresponding author(s): Xi Cheng

Last updated by author(s): May 17, 2024

Reporting Summary

Nature Portfolio wishes to improve the reproducibility of the work that we publish. This form provides structure for consistency and transparency in reporting. For further information on Nature Portfolio policies, see our Editorial Policies and the Editorial Policy Checklist.

Statistics

For all statistical analyses, confirm that the following items are present in the figure legend, table legend, main text, or Methods section.

n/a	Confirmed	
☐	☒	The exact sample size (n) for each experimental group/condition, given as a discrete number and unit of measurement
☐	☒	A statement on whether measurements were taken from distinct samples or whether the same sample was measured repeatedly
☐	☒	The statistical test(s) used AND whether they are one- or two-sided<br>*Only common tests should be described solely by name; describe more complex techniques in the Methods section.*
☒	☐	A description of all covariates tested
☒	☐	A description of any assumptions or corrections, such as tests of normality and adjustment for multiple comparisons
☐	☒	A full description of the statistical parameters including central tendency (e.g. means) or other basic estimates (e.g. regression coefficient) AND variation (e.g. standard deviation) or associated estimates of uncertainty (e.g. confidence intervals)
☐	☒	For null hypothesis testing, the test statistic (e.g. F, t, r) with confidence intervals, effect sizes, degrees of freedom and P value noted<br>*Give P values as exact values whenever suitable.*
☒	☐	For Bayesian analysis, information on the choice of priors and Markov chain Monte Carlo settings
☒	☐	For hierarchical and complex designs, identification of the appropriate level for tests and full reporting of outcomes
☒	☐	Estimates of effect sizes (e.g. Cohen's d, Pearson's r), indicating how they were calculated

Our web collection on statistics for biologists contains articles on many of the points above.

Software and code

Policy information about availability of computer code

Data collection	Data were collected from Protein Data Bank (https://www.rcsb.org). MD simulation used CHARMM-GUI (https://charmm-gui.org/?doc=input/membrane.bilayer) and Amber 20.
Data analysis	The training of model was based on torch 1.13.1 and torch-geometric 2.3.0. Data preprocess was based on rdkit 2022.9.5, biopython 1.81, unicore 0.0.1 and mdanalysis 2.4.2. Sequence cluster was based on MMseqs2 version 2fad714b525f1975b62c2d2b5aff28274ad57466.

For manuscripts utilizing custom algorithms or software that are central to the research but not yet described in published literature, software must be made available to editors and reviewers. We strongly encourage code deposition in a community repository (e.g. GitHub). See the Nature Portfolio guidelines for submitting code & software for further information.

Data

Policy information about availability of data

All manuscripts must include a data availability statement. This statement should provide the following information, where applicable:

- Accession codes, unique identifiers, or web links for publicly available datasets
- A description of any restrictions on data availability
- For clinical datasets or third party data, please ensure that the statement adheres to our policy

All input data used in this study are freely available from PDB (https://www.rcsb.org), AlphaFold (https://alphafold.com) and PubChem (https://pubchem.ncbi.nlm.nih.gov). The training, validation and independent test sets are available for download at https://github.com/xichengeva/DeepGlycanSite/tree/main/datasets.

Research involving human participants, their data, or biological material

Policy information about studies with human participants or human data. See also policy information about sex, gender (identity/presentation), and sexual orientation and race, ethnicity and racism.

Reporting on sex and gender	Not applicable.
Reporting on race, ethnicity, or other socially relevant groupings	Not applicable.
Population characteristics	Not applicable.
Recruitment	Not applicable.
Ethics oversight	Not applicable.

Note that full information on the approval of the study protocol must also be provided in the manuscript.

Field-specific reporting

Please select the one below that is the best fit for your research. If you are not sure, read the appropriate sections before making your selection.

[x] Life sciences [ ] Behavioural & social sciences [ ] Ecological, evolutionary & environmental sciences

For a reference copy of the document with all sections, see nature.com/documents/nr-reporting-summary-flat.pdf

Life sciences study design

All studies must disclose on these points even when the disclosure is negative.

Sample size	For no statistics methods were used to determine the sample size used, a minimum of three samples were analyzed, which were biologically repeated for at least three times.
Data exclusions	In the test set, the sequences which are more than 95% similarity with training set were excluded.
Replication	Each experiment was performed in technical triplicates. Each data was collected from at least three independent experiments, which was also described in the corresponding figure legend.
Randomization	Not used in this study.
Blinding	Not used in this study.

Reporting for specific materials, systems and methods

We require information from authors about some types of materials, experimental systems and methods used in many studies. Here, indicate whether each material, system or method listed is relevant to your study. If you are not sure if a list item applies to your research, read the appropriate section before selecting a response.

Materials & experimental systems

n/a	Involved in the study
☒	☐ Antibodies
☐	☒ Eukaryotic cell lines
☒	☐ Palaeontology and archaeology
☒	☐ Animals and other organisms
☒	☐ Clinical data
☒	☐ Dual use research of concern
☒	☐ Plants

Methods

n/a	Involved in the study
☒	☐ ChIP-seq
☒	☐ Flow cytometry
☒	☐ MRI-based neuroimaging

Eukaryotic cell lines

Policy information about cell lines and Sex and Gender in Research

Cell line source(s)	HEK293 were purchased from ATCC and the identity of the cell line was confirmed by carrying out fingerprinting (Shanghai Genening Biotechnologies Inc, Shanghai, China).
Authentication	Genening Biotechnologies Inc, Shanghai, China.
Mycoplasma contamination	HEK293 cells were tested using the MycoBlue Mycoplasma Detector Kit (Vazyme,China), indicating that the cells were not contaminated by Mycoplasma.
Commonly misidentified lines (See ICLAC register)	No

Plants

Seed stocks	Not applicable.
Novel plant genotypes	Not applicable.
Authentication	Not applicable.
